# Polish Yellow Sweet Clover (*Melilotus officinalis* L.) Honey, Chromatographic Fingerprints, and Chemical Markers

**DOI:** 10.3390/molecules22010138

**Published:** 2017-01-15

**Authors:** Izabela Jasicka-Misiak, Ewa Makowicz, Natalia Stanek

**Affiliations:** Faculty of Chemistry, Opole University, Oleska 48, 45-052 Opole, Poland; makowicz.ewa@gmail.com (E.M.); natalka0703@op.pl (N.S.)

**Keywords:** yellow sweet clover, *Melilotus officinalis*, phenolic, volatile, honey markers

## Abstract

A case study of Polish *Melilotus officinalis* honey was presented for the first time. Gas chromatography–mass spectrometry (GC-MS) (after steam distillation, Soxhlet extraction, ultrasonic solvent extraction, and solid phase extraction (SPE)) and targeted high performance liquid chromatography with a photodiode array detector (HPLC-PAD) were applied to determine the characteristic components of honey. While ubiquitous in most honeys, carbohydrates, terpene derivatives, and phenylacetic acid dominated in the Soxhlet extracts (25.54%) and in the application of SPE (13.04%). In addition, lumichrome (1.85%) was found, and may be considered as a marker of this honey. Due to the presence of these compounds, Polish yellow sweet clover honey is similar to French lavender honeys. The major compounds determined in the methanolic extract were (+)-catechine (39.7%) and gallic acid (up to 30%), which can be regarded as specific chemical markers of the botanical origin of melilot honey. With respect to total phenolic and flavonoid contents, 1,1-diphenyl-2-picrylhydrazyl (DPPH) assays were determined spectrophotometrically. The honey exhibited a moderate antioxidant activity, typical for light honeys, which correlates well with its phenolic and flavonoid composition.

## 1. Introduction

*Melilotus officinalis*, also known as yellow melilot or yellow sweet clover, is a member of the Fabaceae family. It is a biennial aromatic herb, native to Europe and Asia, and has been considered a medicinal plant since ancient times. Hippocrates, the father of medicine, used sweet clover herb to treat skin ulcers, while Dioscorides described melilot as an emollient and anti-edematous drug. Bock recommended melilot for emollient ulcers, abscesses, and as an analgesic agent. The well-known French herbalist, Leclerc, described the sedative and antispasmodic actions of sweet clover [1]. In 19th century medicine, herb and flowering shoots were used to prepare mush compresses, whereas an herbal tea found application as a wash and rinse for swelling and swollen glands, abscesses, and swelling of the lymph nodes. Tea herbs, or the flowers themselves, were applied in cases of cold, mucosity, and respiratory and gastrointestinal catarrh [1].

In modern natural medicine, sweet clover is still used to support the treatment of many diseases. Extracts of this herb, containing coumarins (e.g., 7-hydroxycoumarin and 6,7-dihydroxycoumarin) are used in the treatment of phlebitis, in preventing thrombosis and vascular fragility [2], and in the treatment of varicose veins and hemorrhoids [3]. Sweet clover has sedative and antispasmodic effects towards the smooth muscles of the digestive tract, respiratory, and excretory systems, and also affects the effect of choleretics. *M. officinalis* extract is also used in the treatment of diabetic foot ulcers [2]. In addition to the reported medicinal properties, melilot is also used to add flavor and aroma to foods and beverages [4].

Yellow melilot smells like freshly-mown hay. All species of *Melilotus*, when flowering, have a peculiar sweet odor, which, through drying, becomes stronger and more agreeable. The characteristic sweet odor is derived from its coumarins (e.g., coumarin, 7-hydroxycoumarin and 6,7-dihydroxycoumarin). In Europe, dried *Melilotus* was traditionally placed in storage boxes and chests with clothing and linens in order to protect them from moths, in the same manner as dried lavender [5]. Medical sweet clover herb contains coumarin, which breaks down, when the plant is spoiled or damaged, into dicoumarin. This compound is used as a blood thinner and anticoagulant in rat and mouse poisons, and also for treating human ailments [6].

Recently, scientists and food producers have been more and more interested in plant-based foods [7,8,9]. These generally less-processed foods contain many valuable, biological active substances, with proven beneficial effects on consumers’ health. Among others, bee-derived products, in particular honey, are very highly appreciated and recognized. Honey, beyond its flavor and nutritional value, is also known to have proven therapeutic properties, undoubtedly one of its most valuable qualities. This fact, not only provides many opportunities for the use of honey as an ingredient in many medicinal products, but also increases its value as an element of human diet. Therefore, integrated studies on the chemical composition, botanical origin, and biological activity of bee products are very important in the context of their use as an ingredient in modern food.

Polish *Melilotus* species (yellow and white sweet clover) blooms from June to late August, and are attractive and productive nectariferous plants. However, according to the best of our knowledge, the chemical composition and biological activity of yellow sweet clover honey have not yet been investigated. Therefore, the main objective of this study was to characterize Polish yellow sweet clover honey through investigation of the group of volatile and phenolic compounds that can be isolated from this product. The characterization was based, in each group, on the determination of its chromatographic fingerprint profile, and we attempted to identify the specific chemicals within the profile. Due to substantial differences in physicochemical properties between the two groups, two approaches were used in order to achieve this goal. For volatiles, isolation by either Soxhlet or ultrasonic extraction, as well as through steam distillation in the presence of an organic solvent, combined with a GC-MS analysis, was applied. To characterize a group of phenolic compounds, solid phase extraction was used, followed by analysis of the extract using HPLC-PAD (including the determination of total phenolic contents). Based on the obtained results, coumarin has been selected as a potential marker for this honey, and its content was quantified using GC-MS. Additionally, the determination of antioxidant properties of honey, utilizing DPPH assays, was performed.

## 2. Results and Discussion

### 2.1. Volatile Compounds Identified by GC-MS

GC-MS analysis of dichloromethane extracts, obtained after steam distillation, Soxhlet extraction, and ultrasonic solvent extraction (USE) of three Polish sweet yellow clover honeys led to the identification of 82 volatile compounds. These studies also showed that the applied methods for extraction appear to be suitable procedures for the isolation of volatiles from honey samples. Average peak areas of the identified compounds for honeys obtained from different years are shown in Table 1. Chromatographic analysis of the extracts obtained using the solid phase extraction method, with the use of an SDBL cartridge, enabled the identification of 26 volatile compounds (Table 2). Volatile compounds present in *Melilotus officinalis* honey represent different groups of chemical entities; below we present the average content of the compounds present in the highest concentrations in all tested honey samples. When using the steam distillation technique, the highest contents for β-phellandrene (2.17%), benzenacetaldehyde (5.84%), phenylethyl alcohol (2.03%), β-menthane (2.23%), thymol (2.03%), and ethyl 2-(5-methyl-5-vinyltetrahydrofuran-2-yl)propan-2-ylcarbonate (2.84%) were detected. When Soxhlet extraction was used, the highest contents for carophyllene (11.23%), 13-epimanool (1.45%), phenylacetic acid (5.35%), and *p*-eugenol (6.54%) were observed. While using USE, the predominant compounds were phenylacetic acid (25.54%), *α*-(phenylmethyl)benzenethanol (6.44%), and *p*-acetoxyanisole (2.89%). In the case of the SPE method, two compounds had the largest areas of peaks: Phenylacetic acid (13.04%) and *N*-butylbenzensulfonamide (10.94%). This method, however, provided the lowest number of compounds, and, thus, the results are incomparable with other methods. Coumarin is a volatile compound, which is not widespread among different varieties of honeys and, thus, is probably one of the most interesting compounds identified in sweet clover honey. This substance belongs to a group of naturally-occurring phenolic compounds that contain the characteristic benzo[*α*]pyrone moiety and are produced via the shikimate pathway [10]. Coumarins are found in high levels in several plant species, such as the cinnamon tree, lavender, *Prunus mahaleb* L., and in sweet yellow clover [11,12,13,14]. It has been identified as a compound with very interesting pharmaceutical properties, such as the ability to increase blood flow in veins and decrease capillary permeability. Coumarin also poses antifungal and anti-cancer properties, but can be toxic when it is used at high doses for a long time [10]. The average content of coumarin in two-year-old Polish sweet yellow clover honey is 728 ng/g of honey, with the highest concentration being 902 ng/g of honey (see Table 3). Coumarin was not detected in fresh honey after harvesting (sample from 2016). The greatest amount of coumarin was obtained through steam distillation.

The results of other studies show that coumarin is also present in French lavender honeys, derived from *Lavandula angustifolia* Mill., *Lavendula angustifolia x latifolia*, and *Lavendula hybrid* Reverchon II, where it mainly acts as a freshness indicator, and in honey from *Prunus mahaleb* L., where it serves as a nonspecific biomarker [12,13]. In lavender honeys, the average concentration of coumarin is lower in honeys analyzed after harvest (62 to 292 ng/g) with a progressive increase during storage (from 512 to 1720 ng/g in one-year-old honey) [12,15]. The coumarin concentration in our two-year-old sweet yellow clover honey (2014) is lower than that in two-year-old lavender honey. These analytical data may suggest that the total content of coumarin in lavender honeys is higher than that in Polish sweet yellow clover honeys.

The second interesting compound, which was isolated using the SPE method, is lumichrome. This compound is a common breakdown product of riboflavin, which forms in the presence of light, in neutral or acidic solutions. In an alkaline solution, riboflavin is decomposed into lumiflavin. Other studies suggested that it could also be produced via an active mechanism, and cause different biochemical activities. Lumichrome plays an important regulatory role in plants by enhancing photosynthesis in soybean and corn, and is also probably responsible for seeding development in legumes and cereals. Thus far, there is no evidence that lumichrome has any important biological role in humans [16,17]. Lumichrome, for the first time, was identified in high concentrations in thistle (*Galactites tomentosa* Moench) honey, and, together with phenylacetic acid, was proposed as a marker for this monofloral honey [18]. Further studies have also shown the presence of lumichrome in other honeys: Dalmatian sage and cornflower (*Centaurea cyanus* L.) honeys, as well as a presence in other honeys in lower concentrations (*Aarbutus unedo* L., *Asphodelus microcrapus*, *Citruss* spp*. Eucayptus* spp., *Hedysarum coronarium* L., *Salvia officinalis* L., and *Satureja* spp.). It appears that, for honeys that contain lumichrome, the concentration of phenylacetic acid is higher than the normally observed in honey samples [17,19] with one exception—in *Dalmatian sage* honey, authors have demonstrated the presence of lumichrome, while phenylacetic acid was not found in the tested samples [17]. We have confirmed that lumichrome is present in Polish sweet yellow clover honey (*Melilotus officinalis)* and that it is accompanied by an enhanced concentration of phenylacetic acid. Obtained results suggest that the presence of coumarin in sweet yellow clover honey may be significant for botanical classification of this type of monofloral honey. Additionally, lumichrome and a high concentration of phenylacetic acid can be treated as potential markers.

### 2.2. Phenolic Compounds

One of the aims of this study was to show and compare antioxidant activity, and the phenolic and flavonoids contents of yellow sweet clover honey. To the best our knowledge, chemical composition and antioxidant activity of this branch of honey has not yet been investigated.

#### 2.2.1. Total Phenolic and Flavonoids Content (TPC and TFC)

According to color, honeys are generally divided into dark and light types. The first category includes honeys such as heather, oak, and chestnut, and, indeed, these honeys have the highest content of phenolic compounds compared to the second group, light-colored honeys, such as acacia, rape, goldenrod, and rhododendron, and they have significantly lower contents of phenolic compounds [20,21].

It is well known that the TPC level of honeys exist in a wide range, from 0.5 mg/100 g to 130 mg/100 g of honey. Flavonoids constitute approximately 5% of these phenolic compounds.

The amount of total phenolic content in Polish yellow sweet clover honey was measured using the Folin-Ciocalteu reagent method (Table 4) and was expressed as gallic acid equivalent (GAE). Phenolic content varied from 54.06 mg to 55.51 mg GAE/100 g of honey. The results obtained in the present study also showed that the values are within the ranges obtained for lavender honey (53.39 mg GAE/100 g), lime honey (41.20 mg GAE/100 g) [21], sunflower honey (54.63 mg GAE/100 g), sage honey (55.40 mg GAE/100 g), and goldenrod honey (49.24 mg GAE/100 g) [22].

The TFC of yellow sweet clover honey was expressed as quercetin equivalents and varied from 2.29 mg to 2.39 mg QE/100 g of honey. As above, the TFC is similar to that of lavender honey (2.20 mg QE/100 g) [21].

#### 2.2.2. HPLC-PAD Analysis of Honey Samples

**Figure 1:**
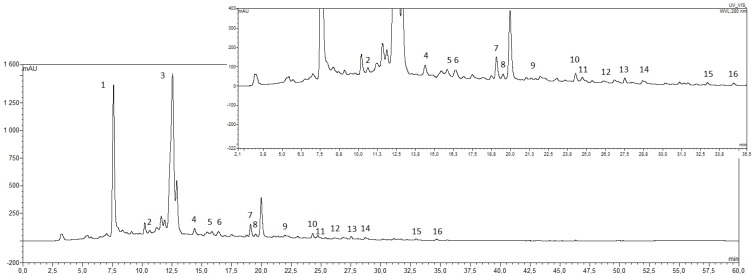
High performance liquid chromatography (HPLC) chromatograms of phenolic compounds in methanolic extracts of Polish yellow sweet clover honeys. Identified compounds are: (1) gallic acid; (2) 3,4-dihydroxybenzoic acid; (3) (+)-catechin; (4) 4-hydroxybenzoic acid; (5) caffeic acid; (6) 3-hydroxybenzoic acid; (7) *p*-coumaric acid; (8) ferulic acid; (9) rosmarinic acid; (10) ellagic acid; (11) myricetin; (12) cinnamic acid; (13) quercetin; (14) genstein; (15) pinocembrin; and (16) morin hydrate.

The HPLC analysis of sweet clover honey samples indicated the presence of 15 phenolic compounds; nine phenolic acids: gallic, 4-hydroxybenzoic, caffeic, 3-hydroxybenzoic, *p*-coumaric, ferulic, rosmarinic, ellagic, and cinnamic; and six flavonoids: (+)-catechin, myricetin, quercetin, genistein, pinocembrin, and morin. A representative HPLC chromatogram recorded at 280 nm is shown in Figure 1.

The calculated levels of individual identified phenolic compounds are shown in Table 4. Targeted HPLC-PAD analysis indicated the presence of significant amounts of gallic acid (23.23 ± 4.52 mg/100 g honey) and (+)-catechin (26.79 ± 2.99 mg/100 g honey).

Phenolic acids represent almost 30.0% of the total identified phenolic compounds present in each of the analyzed samples. The remaining determined compounds were flavonoids at a level of only about 5% of identified phenolic compounds, which is in accordance with the literature data.

All of the identified compounds have been previously found in various monofloral honeys. Gallic acid (1.35 mg to 11.27 mg GAE/100 g) and caffeic acid (0.41 mg to 1.60 mg GAE/100 g) were considered as markers for the floral origin of lavender honey [23]. Much smaller quantities of catechin (3.67 mg GAE/100 g) and *p*-coumaric acid (0.103 mg GAE/100 g) were identified in manuka honey [24]. Gallic acid (0.86 mg GAE/100 g), *p*-coumaric acid (0.30 mg GAE/100 g), ellagic acid (0.56 mg GAE/100 g), and quercetin (15.89 mg GAE/100 g) were found in in gelam honey [25]. It is worth mentioning that the levels of gallic acid or catechin are lower than in yellow sweet clover honey. Additionally, gallic acid and catechin contents are significant if compared to the contents of the other phenolics. Thus, they might be considered as characteristic compounds of the studied honey.

#### 2.2.3. Antiradical Activity (DPPH Test)

Antioxidant activity was measured by using a DPPH-based procedure. Literature data indicate that dark honeys are highly active as radical scavengers, more active than light honeys [26,27]. Additionally, antiradical activity depends on the location of floral sources of honey (botanical origin) [28]. The result of the DPPH test, 55.96%, is similar to that recorded previously for lime (63.48%) and rape honey (55.16%) [27].

Several studies indicate that there is a correlation between the total phenolic and flavonoids content and antiradical activity of honey—the higher content of phenolics results in higher antiradical activity [29,30]. Yellow sweet clover honey contains 67.55 mg of GAE/100 g and 2.35 mg of QE/100 g, while its antiradical activity is 55.96%. This result correlates well with literature data.

## 3. Materials and Methods

### 3.1. Reagents and Honey Samples

All chemicals were of analytical grade. Methanol, dichloromethane, ethanol dibasic sodium phosphate heptahydrate (Na_2_HPO_4_ × 7H_2_O), hydrochloric acid, and 85% phosphoric acid were purchased from POCH S.A. (Gliwice, Poland). Fifteen flavonoids: Apigenin, (+)-catechin, (−)-epicatechin, formononetin, galangin, genistein, kaempferol, chrysin, quercetin, morin hydrate, myricetin, (+)-naringenin, pelargonidin chloride, pinocembrin, and rutin hydrate; as well as 11 phenolic acids: 3-hydroxybenzoic acid, 3,4-dihydroxybenzoic acid, 4-hydroxybenzoic acid, caffeic acid, cinnamic acid, chlorogenic acid, ellagic acid, ferulic acid, gallic acid, *p*-coumaric acid, rosmarinic acid, syringic acid, and vanillic acid, used as standards, were purchased from Sigma-Aldrich (Poznań, Poland). Additionally, sesquiterpene, (±)-abscisic acid, and coumarin (Sigma-Aldrich, Poznań, Poland) were used as standards. Stock solutions of each standard were prepared with HPLC-grade methanol at a concentration of 0.01 mmol/L and were stored at 4 °C with protection from light. Amberlite XAD-2, used as an adsorption resin for the extraction step, was obtained from Supelco (Bellefonte, PA, USA).

This study was carried out with nine yellow sweet clover honeys (*Melilotus officinalis* L.) obtained from professional beekeepers from the southeastern part of Poland (Subcarpathian Voivodeship) in three successive years: 2014, 2015, and 2016. The honey type was declared by the producers, based on the availability of nectar sources, location of the beehives, and sensory characteristics. All of the samples, after acquisition from beekeepers, were stored in hermetically sealed glass jars, in the dark, at 4 °C.

### 3.2. Extraction and Determination of Volatile Compounds

#### 3.2.1. Steam distillation 

For steam distillation, 150 g of a Polish honey sample was dissolved in 150 mL distilled water and transferred into a 500 mL round-bottomed flask. The distillation time was 3 h in every case, and was repeated three times with the same amount of honey. The obtained distillate was transferred into a separation funnel and the aqueous phase was extracted with three portions of dichloromethane (total volume used for extraction was 400 mL). Organic layers were combined and filtered through layers of anhydrous magnesium sulfate. All fractions were filtered through anhydrous magnesium sulfate and concentrated on a rotary evaporator at 35 °C to a final volume of 100 µL, and 1 µL was analyzed using GC-MS. The entire procedure was performed three times for each honey sample.

#### 3.2.2. Soxhlet Extraction 

Fifty-five grams of a honey sample was placed in a cellulose thimble and placed in a Soxhlet extraction apparatus containing 150 mL of dichloromethane and extraction was carried out for 10 h. Obtained samples were filtered through anhydrous magnesium sulfate and concentrated on a rotary evaporator at 35 °C to a final volume of 100 µL, and 1 µL was analyzed using GC-MS. The procedure was performed three times for each sample.

#### 3.2.3. Ultrasound Solvent Extraction (USE) 

USE was performed as described previously [31] with some modifications. Forty grams of Polish sweet yellow clover honey sample were dissolved in 22 mL of distilled water in a 150-mL flask. Magnesium sulphate (1.5 g) was added and each sample was extensively vortexed. Then, 20 mL of dichloromethane was used as the extraction solvent for each honey sample. USE was performed in an ultrasound cleaning bath (Cole-Parmer 8891) at 25 °C for 30 min. After the end of each sonication, the sample was introduced via a separation funnel and 20 mL of a saturated solution of NaCl was added for separation of organic and aqueous phases at room temperature. When the two layers were separated, the emulsion was collected. The whole extract was centrifuged at 3000 rpm, and the organic layer was collected and filtered through anhydrous magnesium sulphate. The aqueous layer was returned to the flask and another batch of 20 mL of dichloromethane was added. Each sample was extracted three times. Obtained fractions were filtered through anhydrous magnesium sulfate and concentrated on a rotary evaporator at 35 °C to a final volume of 100 µL, and 1 µL was analyzed using GC-MS. The entire procedure was carried out in triplicate for each honey sample.

#### 3.2.4. Solid Phase Extraction (SPE) 

SPE was done as described previously [32] with some modifications and was performed in a Baker SPE 12 g vacuum model (J. T. Baker^®^, Phillipsburg, NJ, USA) with a flow rate of 2 mL/min. Strata–SDBL with 200 mg of styrene-divinylbenzene resin cartridges (Phenomenex) were used for analysis. Prior to use, cartridges were conditioned by rinsing with 4 mL of dichloromethane, 4 mL of water, and 4 mL of ethanol–water mixture (12%, *v*/*v*). Then, 15 g of honey sample dissolved in water was rinsed through the cartridges, and, subsequently, sugars and other hydrophilic components were washed with 20 mL of water; finally, elution was performed with 20 mL of dichloromethane. Obtained fractions were filtered through anhydrous magnesium sulfate and concentrated on a rotary evaporator at 35 °C to a final volume of 100 µL, and 1 µL was analyzed by GC-MS. The whole procedure was carried out in triplicate for each sample.

#### 3.2.5. Quantification of Coumarin Content by GC-MS

Concentration of the coumarin in Polish sweet yellow clover honey samples was performed with respect to a standard curve. A standard stock solution of 1 mg/mL of coumarin was prepared in dichloromethane. The calibration curve was constructed for seven different concentrations of coumarin standards, three replications each. The equation used to calculate the amount of coumarin in sweet yellow clover honey samples was set as: y = 1,072,297x − 3,941,898 (R^2^ = 0.994). Repeatability of analyses was assessed using the same analysis under the same analytical conditions.

#### 3.2.6. GC-MS Analysis 

Analyses using gas chromatography-mass spectrometry were carried out with an HP 6890 GC System (Hewlett Packard, Böblingen, Germany) coupled to a 5973 Mass Selective Detector (Hewlett Packard, Böblingen, Germany). The mass detector worked in the electron impact ion-isolation mode at 70 eV, the mass range was 10–600 units, and the ion source temperature was 230 °C. A total of 1 µL of extract was injected into the GC equipped with a 30 m × 0.25 mm i.d. capillary column with an HP-5MS ((5%-phenyl)-methylpolysiloxane, Agilent J and W GC column) with a coating thickness of 0.25 µm. Helium, at a constant flow rate of 2 mL/min, was used as the carrier gas, while chromatographic conditions were set as follows. Oven temperature was initially held at 40 °C for 3 min, and then increased to 180 °C at a rate of 5 °C/min, and, finally, increased to 250 °C at a rate of 10 °C/min. The equilibration time was 0.5 min. The components were identified by comparison of their mass spectra with those of the spectrometer database using the NIST library (National Institute of Standards and Technology, Gaithersburg, MD, USA), with probabilities higher than 80%. Each sample was analyzed three times.

### 3.3. Extraction and Determination of Phenolic Compounds

Phenolic compounds were extracted as described previously [33] with some modifications. Twenty-five grams of honey were dissolved in 125 mL of acidified water (pH = 2 with HCl) and homogenized in an ultrasonic bath for 1 h until a nearly-clear fluid was obtained. The sample was then filtered through filter paper to remove any solid particles. Simultaneously, 40 g of Amberlite XAD-2 (Supelco, Bellefonte, PA, USA, pore size: 9 nm, particle size: 0.3–1.2 mm) was soaked in methanol and stirred with a magnetic stirrer for 30 min. The mixture was allowed to stand for 10 min and was packed into a glass column (2.5 cm × 55 cm). Next, the honey solution was transferred to a glass column and allowed to stand for 30–45 min in order to absorb honey phenolic compounds. The column was washed with acidified water (0.05 M HCl, 250 mL) and then distilled water (300 mL) to remove all sugars and other polar compounds. The flavonoids and phenolic compounds were eluted with methanol (500 mL) and the phenolic fraction was evaporated under reduced pressure at 40 °C. The residue was then dissolved in 2 mL of methanol (HPLC-grade) and analyzed by HPLC with photodiode array (PDA) detection. The solution was evaporated again and the obtained residue was extracted three times with diethyl ether (15 mL). The remains were redissolved in 2 mL methanol (HPLC-grade) and analyzed by HPLC again.

#### 3.3.1. HPLC/PAD Analysis

Analyses of the honey extracts were performed using an Ultimate 3000 Dionex HPLC system with a photodiode array detector (PDA) (Germering, Germany) and a Gemini 5 µm C18 reverse phase column (250 × 4.6 mm, particle size 5 µm). The mobile phase consisted of methanol (solvent A) and 0.01 M aqueous phosphate buffer at pH = 2.5 (solvent B) at a flow rate of 1 mL/min. The gradient separation was performed as follows (A:B ratio): 10%–90% t = 0; 40%–60% t = 13.5 min; 90%–10% t = 39 min; 100%–0% t = 42 min; 10%–90% t = 55 min. The analyses were carried out at 30 °C. The chromatograms were recorded at 214 and 280 nm, since most honey flavonoids and phenolic acids show their UV absorption maxima within the range of these wavelengths. The identification of phenolic compounds present in the honey extracts were based on the comparison of UV spectra and retention times with those found for standard compounds.

#### 3.3.2. Total Phenolic and Flavonoids Content

The Folin-Ciocalteu method was used to spectrophotometrically determine the TPC [27]. Briefly, 5 mL of honey extracts (0.5 g honey/50 mL of distilled water) were mixed with 0.5 mL Folin-Ciocalteu reagent (FC) and 1.5 mL 20% Na_2_CO_3_ solution and were placed into a 10-mL volumetric flask. Then, they were diluted to 10 mL and kept in the dark for 120 min. After this time, absorbance was measured at 760 nm (Spectrophotometer Hitachi U-2810, Tokyo, Japan) against the blank (water). TPC was expressed in terms of milligrams of gallic acid equivalents mg per 100 g honey and was determined as the average from three parallel measurements.

The TFC was determined using a colorimetric method [34]. Briefly, 1 mL of honey extract (50 g honey/50 mL of methanol) was mixed with 1 mL AlCl_3_ solution (10%). The mixture was allowed to stand for 60 min. Absorbance was measured at 420 nm against the blank (mixture of methanol and reagent). Results were expressed as quercetin equivalents in milligrams per 100 g honey, and determined as the average from three parallel measurements.

#### 3.3.3. Antiradical Activity (DPPH Test)

The antiradical activity extract of honey was evaluated using a modified method of Meda et al. [34]. Briefly, 1 mL of methanolic honey extract (10 g honey/50 mL of methanol) was mixed with 4 mL of a methanolic solution of DPPH radicals (0.1 mM). The control test was performed with a methanol-lacking extract. The mixture was mixed and allowed to stand in the dark for 30 min. Absorbance was measured at 517 nm against the blank (methanol). Antiradical activity (%) of the honey samples was calculated using the following formula:

AA [%] = 100 × (Abs_control_ − Abs_sample_)/Abs_control_

where Abs represents the absorbance.

## 4. Conclusions

The HPLC-PAD data of the first investigation of the composition of unifloral *M. officinalis* honey revealed the dominant content of gallic acid and (+)-catechin. Their contents, and relative amounts, in the analyzed honey samples were constant and may be proposed as useful chemical markers of the floral origin of this honey. Furthermore, GC-MS analysis of the honey extracts showed the presence of interesting substances: Pharmacologically-active coumarin, which has, so far, been described as a main ingredient of French lavender honeys, and lumichrome, a derivative of the vitamin riboflavin, suggested as a marker among Dalmatian sage (*S. officinalis* L.) unifloral honey. The results of analysis of the tested honey samples clearly indicate that the combined presence of these compounds, alongside the high level of gallic acid and (+)-catechin (all of these compounds are shown in Figure 1), could be characteristic chemical fingerprints used to distinguish Polish yellow sweet clover honey from other unifloral honeys.

## Figures and Tables

**Table 1:** Volatile compounds of Polish sweet yellow clover honey characterized by gas chromatography–mass spectrometry (GC-MS) in extracts obtained with different procedures.

No.	Compounds	Peak Area (%)								
		A			B			C		
		H14	H15	H16	H14	H15	H16	H14	H15	H16
		Av. (*n* = 3)			Av. (*n* = 3)			Av. (*n* = 3)		
1	(1*S*,15*S*)-Bicyclo[13.1.0]hexadecane	-	-	-	0.00 ± 0.00	0.69 ± 0.09	0.00 ± 0.00	-	-	-
2	*β*-Phellandrene	1.96 ± 0.21	0.61 ± 0.28	1.36 ± 0.45	-	-	-	-	-	-
3	*δ*-Selinene	0.10 ± 0.02	0.12 ± 0.05	0.10 ± 0.05	-	-	-	-	-	-
4	11-Tricosene	-	-	-	0.92 ± 0.00	0.92 ± 0.20	1.00 ± 0.31	-	-	-
5	17-Pentatriacontene	-	-	-				0.70 ± 0.23	0.59 ± 0.03	0.56 ± 0.12
6	Docosene	0.51 ± 0.03	0.56 ± 0.04	0.52 ± 0.03	0.96 ± 0.05	1.56 ± 0.05	1.60 ± 0.12	0.00 ± 0.00	1.48 ± 0.63	1.12 ± 0.27
7	1-Heptacosanol	-	-	-	-	-	-	1.46 ± 0.65	1.70 ± 0.04	1.51 ± 0.63
8	1-Heptacosane	-	-	-	-	-	-	1.06 ± 0.26	0.55 ± 0.21	0.59 ± 0.06
9	1-Hexacosene	0.71 ± 0.09	0.80 ± 0.05	0.69 ± 0.10	-	-	-	-	-	-
10	13-Epimanool	-	-	-	0.00 ± 0.00	2.15 ± 0.98	1.46 ± 0.41	-	-	-
11	1-Nonadecene	0.25 ± 0.02	0.25 ± 0.01	0.26 ± 0.01	-	-	-	-	-	-
12	1-Oxa-spiro[4.5]deca-6,9-diene-2,8-dione	0.32 ± 0.10	0.28 ± 0.01	0.28 ± 0.03	-	-	-	-	-	-
13	2,6,10,14-Tetramethyl-7-(3-methylpent-4-enylidene) pentadecane	-	-	-	0.96 ± 0.00	1.19 ± 0.00	0.00 ± 0.00	-	-	-
14	2-Ethyl-5-n-propylphenol	-	-	-	0.23 ± 0.06	0.27 ± 0.16	0.00 ± 0.00	-	-	-
15	2-Ethylhexyl trans-4-methoxycinnamate	0.51 ± 0.02	0.48 ± 0.05	0.00 ± 0.00	-	-	-	-	-	-
16	2-Phenyl-3-(2-furyl)-propenal	0.18 ± 0.01	0.19 ± 0.01	0.19 ± 0.00	-	-	-	-	-	-
17	3,5-di-tert-Butyl-4-hydroxybenzaldehyde	0.00 ± 0.00	0.09 ± 0.01	0.10 ± 0.01	-	-	-	-	-	-
18	3-Furanmethanol/2-Furamethanol *	0.41 ± 0.02	0.35 ± 0.10	0.39 ± 0.20	-	-	-	-	-	-
19	5-Hydroxymethylfurfural	5.94 ± 1.20	2.01 ± 0.01	1.12 ± 0.09	5.51 ± 1.63	4.32 ± 1.97	2.21 ± 0.69	15.21 ± 4.21	13.21 ± 3.28	10.26 ± 1.96
20	(*E*)-9-Octadecenoic acid **	6.98 ± 1.02	7.00 ± 1.25	5.25 ± 0.96	0.96 ± 0.36	1.80 ± 0.64	1.46 ± 0.26	1.64 ± 0.23	3.60 ± 0.76	2.01 ± 0.36
21	(*Z*)-9-Tricosene,	1.26 ± 0.69	1.39 ± 0.78	1.33 ± 0.20	-	-	-	0.90 ± 0.23	0.89 ± 0.41	0.65 ± 0.09
22	Acetophenone	0.00 ± 0.00	0.13 ± 0.08	0.19 ± 0.10	-	-	-	-	-	-
23	Behenic alcohol	-	-	-	0.78 ± 0.22	1.03 ± 0.12	0.00 ± 0.00	-	-	-
24	Benzaldehyde	0.19 ± 0.06	0.25 ± 0.08	0.21 ± 0.42	-	-	-	-	-	-
25	4-Octyl-*N*-(4-octylphenyl)benzenamine	-	-	-	1.23 ± 0.13	2.03 ± 0.29	1.63 ± 0.09	-	-	-
26	Benzeneacetaldehyde	5.69 ± 0.17	6.02 ± 0.10	5.50 ± 0.17	-	-	-	-	-	-
27	Phenylacetic acid	-	-	-	4.13 ± 0.06	6.14 ± 0.65	5.23 ± 0.91	28.12 ± 3.74	26.21 ± 2.78	24.26 ± 3.65
28	*α*-(Phenylmethyl)benzeneethanol	-	-	-	-	-	-	5.59 ± 1.61	8.80 ± 0.09	6.01 ± 1.23
29	Benzoic acid	-	-	-	-	-	-	1.15 ± 0.07	0.85 ± 0.37	1.06 ± 0.33
30	4-Hydroxy-3,5-dimethoxybenzoic acid	-	-	-	-	-	-	1.78 ± 0.23	1.85 ± 0.64	1.79 ± 0.36
31	Benzothiazole	1.21 ± 0.19	1.32 ± 0.09	1.30 ± 0.09	-	-	-	-	-	-
32	Benzyl alcohol	0.25 ± 0.02	0.42 ± 0.10	0.40 ± 0.03	-	-	-	-	-	-
33	2-Methylene4,8,8-trimethyl-4-vinyl-bicyclo[5.2.0]nonane	-	-	-	0.96 ± 0.63	2.93 ± 0.23	2.21 ± 0.09	-	-	-
34	Bis(2-ethylhexyl) maleate	-	-	-	1.01 ± 0.56	1.53 ± 0.23	1.26 ± 0.09	-	-	-
35	Caryophyllene	-	-	-	13.21 ± 3.31	12.26 ± 0.91	13.60 ± 3.69	-	-	-
36	*cis*-13-Octadecenoic acid/*trans*-13-Octadecenoic acid **	-	-	-	1.71 ± 0.63	2.00 ± 0.06	1.96 ± 0.06	-	-	-
37	Coumarin	1.41 ± 0.15	0.98 ± 0.09	0.00 ± 0.00	0.22 ± 0.12	0.19 ± 0.09	0.00 ± 0.00	-	-	-
38	Cyclohept-4-enone	0.21 ± 0.04	0.19 ± 0.10	0.15 ± 0.10	-	-	-	-	-	-
39	*β*-Menthane	1.85 ± 0.35	2.61 ± 0.15	2.52 ± 0.31	-	-	-	-	-	-
40	Cyclopentadecane	-	-	-	0.65 ± 0.26	0.95 ± 0.06	0.71 ± 0.20	-	-	-
41	Docosane	-	-	-	1.36 ± 0.23	1.60 ± 0.16	1.09 ± 0.21	-	-	-
42	Dodecanoic acid	0.38 ± 0.21	0.28 ± 0.08	0.00 ± 0.00	-	-	-	-	-	-
43	*E*-15-Heptadecenal	0.41 ± 0.20	0.38 ± 0.09	0.53 ± 0.20	-	-	-	-	-	-
45	Eicosane	2.65 ± 0.43	3.45 ± 0.45	4.41 ± 1.01	0.87 ± 0.61	1.31 ± 0.26	1.16 ± 0.31	1.86 ± 0.71	1.90 ± 0.06	1.74 ± 0.21
46	Eicosane/Hexadecan/Octadecane *	1.62 ± 0.98	1.76 ± 0.02	1.82 ± 0.63	0.59 ± 0.27	0.91 ± 0.24	0.70 ± 0.36	3.28 ± 1.21	3.56 ± 1.96	0.00 ± 0.00
47	Ethyl 2-(5-methyl-5-vinyltetrahydrofuran-2-yl)propan-2-yl carbonate	2.61 ± 0.13	2.91 ± 0.65	2.79 ± 0.09	1.03 ± 0.52	1.44 ± 0.09	1.26 ± 0.47	-	-	-
48	Ethyl oleate	4.91 ± 0.96	6.21 ± 1.21	5.81 ± 1.12	-	-	-	2.00 ± 0.57	1.68 ± 0.03	1.80 ± 0.07
49	Furfural	2.09 ± 0.21	1.95 ± 0.09	1.21 ± 0.51	-	-	-	-	-	-
50	Heneicosane	2.09 ± 0.20	1.61 ± 0.51	1.82 ± 0.91	-	-	-	0.98 ± 0.14	1.06 ± 0.08	0.50 ± 0.12
51	Hentriacontane	-	-	-	-	-	-	0.80 ± 0.36	0.77 ± 0.21	0.80 ± 0.46
52	Heptacosyl acetate	-	-	-	-	-	-	1.69 ± 0.41	0.00 ± 0.00	1.56 ± 0.98
53	Heptadecane	3.70 ± 0.61	4.42 ± 0.91	4.02 ± 0.98	-	-	-	-	-	-
54	Heptadecane/Hexacosane	-	-	-	-	-	-	2.21 ± 0.97	1.98 ± 0.06	2.04 ± 0.14
55	Methyl hexadecanoate	0.13 ± 0.09	0.00 ± 0.00	0.00 ± 0.00	-	-	-	-	-	-
56	Bis(2-ethylhexyl) hexanedioate	-	-	-	3.36 ± 0.61	6.05 ± 0.78	4.90 ± 1.76	1.41 ± 0.36	2.06 ± 0.26	2.01 ± 0.96
57	Humulene	-	-	-	0.63 ± 0.23	0.95 ± 0.07	0.77 ± 0.31	-	-	-
58	Methyl dehydroabietate	0.61 ± 0.36	0.00 ± 0.00	0.21 ± 0.00	0.96 ± 0.09	1.16 ± 0.45	1.06 ± 0.03	0.00 ± 0.00	0.35 ± 0.21	0.00 ± 0.00
59	n-Hexadecanoic acid	-	-	-	2.79 ± 0.81	4.00 ± 1.15	3.01 ± 0.05	2.90 ± 0.21	2.86 ± 0.06	1.16 ± 0.31
60	Nonacosane	-	-	-	2.39 ± 0.27	2.78 ± 0.97	2.61 ± 0.61	0.91 ± 0.06	1.00 ± 0.09	0.90 ± 0.36
61	Nonadecane	0.20 ± 0.05	0.15 ± 0.06	0.35 ± 0.12	0.37 ± 0.21	0.49 ± 0.08	0.41 ± 0.23	-	-	-
62	9-Methylnonadecane,	-	-	-	1.00 ± 0.05	1.70 ± 0.07	1.40 ± 0.61	-	-	-
63	Nonadecyl trifluoroacetate	-	-	-	0.41 ± 0.26	0.90 ± 0.14	0.85 ± 0.16	1.44 ± 0.02	0.71 ± 0.21	1.01 ± 0.36
64	Octacosane	0.91 ± 0.32	1.26 ± 0.09	1.09 ± 0.26	0.65 ± 0.25	0.80 ± 0.04	0.23 ± 0.10			
65	Octadecane	0.98 ± 0.21	1.79 ± 0.09	1.38 ± 0.31	1.29 ± 0.36	1.46 ± 0.31	0.00 ± 0.00	1.35 ± 0.41	2.36 ± 0.07	1.70 ± 0.36
66	Octadecanoic acid	-	-	-	2.13 ± 1.71	2.50 ± 0.23	2.41 ± 0.96	-	-	-
67	Oleic acid	-	-	-	0.00 ± 0.00	1.53 ± 0.69	1.49 ± 0.27	-	-	-
68	*p*-Acetoxyanisole	-	-	-				0.00 ± 0.00	3.21 ± 1.25	2.01 ± 0.09
69	*p*-Cymen-8-ol	0.00 ± 0.00	0.21 ± 0.09	0.35 ± 0.03	-	-	-	-	-	-
70	*p*-Eugenol	-	-	-	5.65 ± 1.21	7.20 ± 2.26	6.20 ± 0.91	2.26 ± 0.78	1.96 ± 0.21	2.06 ± 0.67
71	2-Methoxy-4-(1-propenyl)phenol	-	-	-	0.56 ± 0.07	0.63 ± 0.12	0.59 ± 0.12	2.81 ± 1.21	3.60 ± 0.94	3.04 ± 0.41
72	Phenylethyl alcohol	1.91 ± 0.21	0.26 ± 0.05	2.01 ± 0.12	-	-	-	-	-	-
73	Supraene/Squalene *	0.81 ± 0.36	1.28 ± 0.14	1.13 ± 0.09	2.71 ± 1.21	2.80 ± 0.08	2.67 ± 0.08	-	-	-
74	Tetracosane	-	-	-				-	-	-
75	Tetradecanoic acid	0.21 ± 0.10	0.98 ± 0.40	0.41 ± 0.21	0.66 ± 0.12	1.20 ± 0.36	0.96 ± 0.23	-	-	-
76	Tetradecyl trifluoroacetate	-	-	-	0.49 ± 0.09	0.50 ± 0.31	0.49 ± 0.09	-	-	-
77	Thymol	2.76 ± 0.09	2.61 ± 0.12	2.85 ± 0.01	-	-	-	-	-	-
78	Tricosane	-	-	-	0.98 ± 0.23	1.65 ± 0.07	1.12 ± 0.36	-	-	-
79	2,3,4,5-Tetramethyl-tricyclo[3.2.1.02,7]oct-3-ene	0.12 ± 0.06	0.25 ± 0.06	0.18 ± 0.09	-	-	-	-	-	-
80	*Z*-10-Tetradecen-1-ol acetate	-	-	-	0.33 ± 0.09	0.48 ± 0.06	0.40 ± 0.20	-	-	-
81	*Z*-12-Pentacosene	0.20 ± 0.09	0.79 ± 0.20	0.71 ± 0.36	1.12 ± 0.23	0.69 ± 0.02	0.60 ± 0.01	-	-	-
82	*α*-Terpinolene	0.00 ± 0.00	0.42 ± 0.00	0.00 ± 0.00	-	-	-	-	-	-

A—steam distillation, B—Soxhlet extraction, C—USE; * Tentatively identified; ** Correct isomer not identified; - Not identified; Av. = average; H14 = honey sample from 2014, H15 = honey sample from 2015, H16 = honey sample from 2016.

**Table 2:** Volatile compounds identified by GC-MS in extracts obtained using solid phase extraction (SPE).

No.	Compounds	Peak Area (%)		
		H14	H15	H16
		Av. (*n* = 3)	Av. (*n* = 3)	Av. (*n* = 3)
1	*trans*-Linallol oxide/*cis*-Linallol oxide **	0.33 ± 0.02	0.00 ± 0.00	0.28 ± 0.04
2	Phynylethylalcohol	0.10 ± 0.03	0.16 ± 0.06	0.00 ± 0.00
3	Hotrienol	0.20 ± 0.09	0.08 ± 0.02	0.00 ± 0.00
4	*p-*cymen-8-ol	0.16 ± 0.09	0.48 ± 0.2	0.09 ± 0.01
5	Benzoic acid	0.00 ± 0.00	0.82 ± 0.09	0.12 ± 0.09
6	Phenylacetic acid	6.21 ± 1.21	19.21 ± 0.21	17.65 ± 2.24
7	*α*-Methylbenzeneethanol	0.19 ± 0.09	3.2 ± 0.91	4.75 ± 1.21
8	Ethyl 4-ethoxybenzoate	0.35 ± 0.09	0.51 ± 0.2	0.63 ± 0.31
9	*N-*Butylbenzenesulfonamide	9.64 ± 0.06	12.06 ± 1.25	12.65 ± 4.52
10	9-Hexadecanoic acid	0.96 ± 0.23	0.00 ± 0.00	1.17 ± 0.42
11	Estra-1,3,5(10)-trien-17-ol	0.96 ± 0.25	3.12 ± 1.09	2.59 ± 0.05
12	Oleic acid	1.83 ± 0.06	1.90 ± 0.36	2.01 ± 0.08
13	Octadecanoic acid/*cis*-13-Octadecenoic acid/*cis*-Vaccenic acid *^/^**	1.45 ± 0.91	1.85 ± 0.03	1.90 ± 0.35
14	1-Docosene	2.08 ± 0.31	2.40 ± 0.39	1.99 ± 0.03
15	(*Z*)-9-Tricosene	0.75 ± 0.03	0.89 ± 0.21	0.72 ± 0.01
16	1-Heptatriacotanol	0.00 ± 0.00	0.84 ± 0.02	0.00 ± 0.00
17	2-Ehylhexyl 3-(4-methoxyphenyl)-2-propenoate	0.71 ± 0.09	0.71 ± 0.21	0.69 ± 0.06
18	Methyl dehydroabietate	0.84 ± 0.09	0.51 ± 0.10	0.79 ± 0.05
19	(*Z*)-9-Octadecenamide	1.81 ± 0.36	3.25 ± 0.51	2.25 ± 0.95
20	1,7,11-Trimethyl-4-(1-methylethyl)-cyclotetradecane	0.00 ± 0.00	0.81 ± 0.32	0.00 ± 0.00
21	Pentacosane	0.90 ± 0.21	1.10 ± 0.01	1.01 ± 0.32
22	Nonadecane	0.70 ± 0.01	1.29 ± 0.21	0.75 ± 0.01
23	Lumichrome	1.82 ± 0.30	1.89 ± 0.09	1.86 ± 0.15
24	Squalene	1.43 ± 0.09	1.62 ± 0.21	1.59 ± 0.21
25	Nonadecyl trifluoroacetate	0.39 ± 0.21	0.51 ± 0.09	0.49 ± 0.19
26	Eicosane	0.48 ± 0.23	2.18 ± 0.23	1.33 ± 0.91

* Tentatively identified; ** Correct isomer not identified; Av. = average; H14 = honey sample from 2014, H15 = honey sample from 2015, H16 = honey sample from 2016.

**Table 3:** Coumarin content in Polish sweet yellow clover honey.

Method	R^2^	Content of Coumarin								
		ng/g of Honey								
		H14			H15			H16		
		Min	Max	Avg	Min	Max	Avg	Min	Max	Avg
Steam distillation	0.994	491.79	902.79	728.66	339.82	629.61	501.23	Not detected		

Min, Max, Avg = minimal, maximal, average concentration of coumarin in honey samples; H14 = honey sample from 2014, H15 = honey sample from 2015, H16 = honey sample from 2016.

**Table 4:** The level of individual identified phenolic compounds (mg/100 g of product) and content of total phenolics (GAE—gallic acid equivalent) and flavonoids. (QE—quercetin equivalent) compounds in Polish yellow sweet clover honeys (methanolic extract).

Phenolic Compounds	GA	C	4-HBA	CA	3-HBA	*p*-CA	FA	RA	EA	MC	CiA	QC	G	P	MH	Total Phenolic Content (mg GAE/100 g) *n* = 2	Total Flavonoids Content (mg QE/100 g) *n* = 2	Radical Scavenging Activity DPPH (%) *n* = 2
Levels of Individual Identified Compounds of Tested Honeys (mg/100 g of Product) (RSD ≤ 5%, *n* = 3)																		
mean	23.23	26.79	0.68	0.41	0.32	1.46	0.25	0.19	0.48	0.36	0.16	0.28	0.26	0.11	0.13	67.55	2.29	55.96
±SD	4.52	2.99	0.43	0.11	0.02	0.12	0.03	0.04	0.03	0.05	0.01	0.03	0.07	0.01	0.03	7.51	0.11	2.35

GA—gallic acid, C—(+)-catechin, 4-HBA—4-hydroxybenzoic acid, CA—caffeic acid, 3-HBA—3-hydroxybenzoic acid, *p*-CA—*p*-coumaric acid, FA—ferulic acid, RA—rosmarinic acid, EA—ellagic acid, MC—myricetin, CiA—cinnamic acid, QC—quercetin, G—genistein, P—pinocembrin, MH—morin hydrate (mean values from three repetitions).
